# Foreword – why this research topic matters: timely imperatives for aeromedical readiness in next-generation aviation operations

**DOI:** 10.3389/fphys.2026.1943318

**Published:** 2026-10-06

**Authors:** Joan Saary, Paul Burke

**Affiliations:** 1 Division of Occupational Medicine, Department of Medicine, University of Toronto, Toronto, ON, Canada; 2 Canadian Forces Environmental Medicine Establishment, Toronto, ON, Canada; 3 Canadian Forces Health Services (CFHS), Ottawa, ON, Canada

**Keywords:** advanced aircraft systems, aerospace physiology, allied interoperability, human performance, integrated readiness

The pace of technological innovation often exceeds expectations. For example, historically in the semi-conductor industry “Moore’s Law” was used to set long-term research and development goals, with forecasts dating back to the 1960’s suggesting yearly doubling in rapid innovation cycles of circuit technology, and thus the semiconductor industry as a whole (Moore, 1965). The primary negative consequence of such a pace of technological change is rapid obsolescence and accordingly high maintenance costs. For situations in which security and survivability of hardware or data are paramount, or in which resources are limited, such brisk obsolescence often poses obstacles to smooth or continued operations (Sandborn, 2008).

Currently, evolutionary biology is also at a cusp of exponential advancement, similar to that of circuit technology decades ago. The brain was once thought to be a black box, and although the secrets of the human brain are still largely hidden, we are now at a turning point in evolutionary neurobiology. The advent of a range of complementary, innovative new measurement methods and technologies (e.g. *in vitro* models) are enabling increasingly complex modeling and evolutionary discoveries (Lancaster, 2024).

The intersection of human capabilities with technology has a multitude of translational benefits. Unravelling challenges in this context can lead to benefits for human preparation, protection, and enhanced performance. However, accelerated technological evolution also challenges the human capability to adapt. Predictions about technological innovation may not take into consideration human-machine teaming and the attendant performance impacts on the human user. Human-machine teaming is the collaborative partnership between humans and autonomous or AI-driven systems, designed to combine human judgment, creativity, and adaptability with the speed, accuracy, and computational power of machines. The goal is to create a synergistic relationship where the whole surpasses the sum of its parts.

The rapid evolution of both innovative technology and capabilities to investigate human cognition and brain health represents an “intersection of need.” This supplement is therefore focussed on human capabilities and physiology specifically in the context of advancing technology in the aviation realm, and the related gaps in our understanding. As various nations, including Canada, transition to emerging generations of advanced aircraft and systems, deep understanding of much more than the aircraft’s performance alone will be essential. The future will require additional focus on the effective ongoing preparation, protection, and enhanced performance of the human operator within an increasingly complex system of systems. This Research Topic is both timely and imperative as it addresses some of the issues in this complex research milieu.

The introduction of next-generation jets represents far more than an aircraft replacement. It reflects a broader transformation: these future airframes are not merely aircraft in the traditional sense; they are highly integrated systems that combine stealth, sensor fusion, advanced mission computing, high-G maneuverability, and growing levels of automation and decision support. Within this environment, modern pilots are required to function in dense, fast-moving, information-rich environments, managing competing streams of data while making time-critical decisions under conditions of extraordinary physiological and cognitive demands. The aviator is not simply flying the aircraft; they are operating as an essential human component of a sophisticated networked system.

These demands carry important implications for operational readiness, mission effectiveness, and long-term personnel sustainment. They also reinforce a central truth of capability development: Advanced aircraft alone do not guarantee advantage. Rather, that advantage depends on the readiness, resilience, and performance of the people who employ them.

This is especially significant when human resources are finite and highly specialized. Each trained pilot represents a major institutional investment in recruitment, training, development, and leadership. Protecting and optimizing those human resources is therefore not only a matter of aircrew health and safety, but of relevance to the team as a whole. As organizations modernize, ensuring that our understanding of the human dimension evolves in parallel with advances in aircraft performance, mission systems, and environmental complexity is evermore relevant.

Researchers are confronted with a variety of questions as a result. How do we enhance pilot performance and resilience in increasingly demanding human-machine environments? How do we detect and mitigate cognitive overload, sensory degradation, fatigue, and physiological strain before they affect flight safety? How do we incorporate physiological monitoring, biomarker-informed surveillance, adaptive training, and predictive analytics into aviation in ways that are evidence-based, interoperable, and useful to leadership? These are all matters of collective readiness.

This Research Topic contributes directly to that objective. The collection is intentionally broad, spanning topics such as aerospace physiology, operational medicine, cognitive science, human factors, and emerging monitoring technologies. Such breadth helps advance a more common understanding of the human challenges associated with next-generation aviation operations. It also supports the development of knowledge that can strengthen doctrine, inform training systems, improve health protection, and promote greater harmonization across aviation communities.

In today’s environment, success depends on shared standards, shared understanding, and enhanced mutual reliance across integrated organizations. Our effectiveness depends on the ability to train together, deploy together, exchange data and lessons learned, and operate with confidence across common frameworks for readiness, human performance, and risk mitigation. In that sense, research on the health and performance of aircrew is itself an important enabler of integrated readiness and allied interoperability.

## Data Availability

The original contributions presented in the study are included in the article/supplementary material. Further inquiries can be directed to the corresponding author.
