# Correction: Foreword – why this research topic matters: timely imperatives for aeromedical readiness in next-generation aviation operations

**DOI:** 10.3389/fphys.2026.2034125

**Published:** 2026-10-28

**Authors:** Frontiers Production Office

**Affiliations:** Frontiers Media SA, Lausanne, Switzerland

**Keywords:** advanced aircraft systems, aerospace physiology, allied interoperability, human performance, integrated readiness

Affiliation 3 “Canadian Forces Health Services (CFHS), Ottawa, ON, Canada” was erroneously given as “Royal Canadian Air Force, Ottawa, ON, Canada”.

The original version of this article has been updated.

